# Interaction Effects Between Tongue-Rolling Behavior and Chronic Stress on Plasma Immune–Inflammatory Indicators, Milk Protein Composition, and Milk Proteome in Dairy Cows

**DOI:** 10.3390/vetsci13020134

**Published:** 2026-01-29

**Authors:** Chenyang Li, Xiaoyang Chen, Tingting Fang, Jie Gao, Guangyong Zhao, Xianhong Gu

**Affiliations:** 1 State Key Laboratory of Animal Nutrition and Feeding, Institute of Animal Science, Chinese Academy of Agricultural Sciences, Beijing 100193, China; chenyang960521@gmail.com (C.L.); bmwa123690@163.com (X.C.); fttftting@163.com (T.F.); gaojie01@caas.cn (J.G.); 2 College of Animal Science and Technology, China Agricultural University, Beijing 100193, China; zhaogy@cau.edu.cn

**Keywords:** animal welfare, dairy cows, stereotypic behaviors, milk protein, immune function, inflammatory response

## Abstract

Tongue-rolling behavior (TR) is commonly observed in dairy cows and is considered a stereotypic behavior indicative of compromised welfare. Chronic stress can impair lactation and immune function, yet the interaction between behavior (TR vs. normal behavior (NB)) and chronic stress (high vs. low) remains unclear. In this study, hair cortisol concentration (HCC) was used to assess stress levels in cows. The cows were first classified into high- and low-stress cows using K-means clustering. Subsequently, cows exhibiting high levels of TR and those exhibiting NB (i.e., no stereotypic behaviors) were selected from both stress categories to establish four groups (*n* = 8 per group): high-stress TR (HT), high-stress NB (HN), low-stress TR (LT), and low-stress NB (LN). We analyzed milk protein composition, milk proteome, and plasma immune-inflammatory indicators. Behavior (TR vs. NB) and chronic stress (high vs. low) showed significant interaction effects on plasma tumor necrosis factor-α (*p* = 0.046), interleukin-6 (*p* = 0.002), and proteomic profiles, involving multiple guanosine triphosphate-binding proteins (*p* < 0.05), transferrin (*p* = 0.001), and complement factors (*p* < 0.05). In addition, TR cows had significantly lower levels of αs1-casein (*p* = 0.019), β-casein (*p* < 0.001), κ-casein (*p* = 0.016), lactoferrin (*p* = 0.003), and plasma immunoglobulin A (*p* = 0.002). These results indicate that, under different chronic stress levels, TR cows differ markedly from NB cows in milk protein expression, immune function, and inflammatory responses. Moreover, milk from TR cows showed reduced quality, and immune dysfunction and inflammation were exacerbated under high stress. Overall, this study provides new insights into the physiological consequences of stereotypic behavior in dairy cows. These findings may help dairy farmers identify cows exhibiting TR as at risk of reduced milk quality and immune dysfunction, allowing for early management interventions to improve animal welfare and productivity.

## Simple Summary

Some dairy cows develop a repetitive tongue-rolling behavior due to frustrated feeding or rumination motivation, which is often seen as a sign of poor welfare. At the same time, cows that live under long-term stress may have weaker immune systems and produce milk of lower quality. However, it is still unclear how this stereotypic behavior and stress influence each other and affect the cow’s health. In this study, we measured the level of cortisol in the hair of cows to reflect their chronic stress and compared tongue-rolling cows and normal behavior cows. We examined their blood and milk to look for signs of inflammation, immune function, and changes in milk proteins. The cows with tongue-rolling had lower levels of protective proteins in their milk and weaker immune indicators in their blood. They also showed stronger signs of inflammation, especially under high stress. These results suggest that cows with tongue-rolling behavior may suffer from both physical and emotional stress, leading to poorer milk quality. Understanding this link can help farmers and researchers improve the living conditions and welfare of dairy cows, leading to healthier animals and better-quality dairy products.

## 1. Introduction

Stereotypic behaviors are repetitive, persistent, and seemingly functionless actions observed in farm animals, zoo animals, and wildlife [1]. Among these, tongue-rolling (TR) is the most common and representative stereotypic behavior in dairy cows, typically characterized by repetitive rolling or extension of the tongue inside or outside the open mouth while the cow is neither eating nor ruminating [2,3]. In intensive farming systems, feed is typically provided by humans. On the one hand, cows often complete their feed intake in a relatively short period, yet they sustain a high motivation to forage [4,5]. On the other hand, a higher proportion of concentrate in the diet leads to reduced time spent eating and ruminating, which also maintains the cows’ motivation for these behaviors at a high level [6]. Consequently, cows may express TR behavior as a mechanism to satisfy their intrinsic foraging needs [1,2,3]. Although TR is frequently observed in intensively housed dairy cows, its behavioral significance and physiological underpinnings remain controversial [2,3,7,8]. Some studies have indicated that TR may reflect underlying metabolic abnormalities and heightened inflammatory activity in dairy cows, and may even be associated with reduced production performance [2,3]. Other studies have shown that cows with TR exhibit altered rumen fermentation profiles and higher production performance [7,8]. Stereotypic behaviors have long been considered reflective of poor animal welfare [9].

Sustained exposure to inadequate welfare conditions is frequently associated with the development of chronic stress in animals [9]. Stress is a major physiological factor influencing metabolic and immune function in dairy cattle. Under chronic stress conditions, the hypothalamic–pituitary–adrenal (HPA) axis is activated, leading to increased cortisol secretion [10]. Prolonged exposure to chronic stress can disrupt endocrine balance, impair immune function, and reduce production performance in dairy cattle [11,12]. In recent years, hair cortisol concentration (HCC) has emerged as a robust and non-invasive biomarker for assessing chronic stress in dairy cows [13,14,15,16,17,18]. Unlike acute measures, HCC integrates cortisol secretion over extended periods, providing a stable and cumulative record of the animal’s long-term physiological stress status [12,19]. Adult Holstein cows grow hair at a rate of approximately 0.6 to 1 cm per month, with complete shedding occurring every three months [20]; thus, a 2 to 4 cm hair sample reflects roughly three months of cortisol accumulation [19]. Hair sampling is non-invasive [21], painless [22], easy to perform [23], and can be stored at room temperature [24], with cortisol levels being relatively insensitive to acute fluctuations [25]. However, HCC can be influenced by the sampling site, and consistency within a study is essential. Current evidence indicates that the tail is the optimal site for hair sampling in dairy cows. Several studies have shown that HCC correlates with the stress status of cows; for example, Comin et al. [15] reported that cows suffering from diseases such as mastitis or metritis exhibited higher HCC compared to healthy cows, and HCC also increases during pregnancy [26].

Milk is an important dietary component for humans and possesses high nutritional value [27]. With the continued growth of the global population, public attention toward milk quality have increased. Milk protein is a key indicator of both milk quality and nutritional value, and its synthesis is influenced by genetic, nutritional, physiological, and environmental factors [28,29]. Dairy milk proteins mainly consist of caseins and whey proteins [30]. Caseins are the predominant proteins in bovine milk, accounting for approximately 80% of total milk protein, and mainly include αs1-casein (αs1-CN), αs2-casein (αs2-CN), β-casein (β-CN), and κ-casein (κ-CN) [31]. Whey proteins primarily include α-lactalbumin (α-LA), β-lactoglobulin (β-LG), lactoferrin (LF), and other minor components [30]. In recent years, increasing attention has been given to non-nutritional factors such as dairy cow behavior and chronic stress, which play important roles in production performance and overall health [7,11].

Although both TR and chronic stress have important implications in dairy production, few studies have systematically investigated whether an interaction exists between TR and chronic stress, or how such an interaction may influence physiological functions such as milk synthesis and immune responses. Furthermore, our previous study found that cows exhibiting high levels of TR had significantly lower milk protein percentage, crude protein percentage, and true protein percentage compared with cows without stereotypic behavior [2]. Therefore, this study continued to use this cohort of cows as subjects and employed a two-factor experimental design, with behavior (TR vs. normal behavior (NB)) and chronic stress (high vs. low) level as the main factors, to evaluate their interactive effects on milk proteome, milk protein composition, and plasma immune-inflammatory indicators. By integrating physiological, behavioral, and biochemical data, this study aims to enhance the understanding of the biological basis of stereotypic behavior in dairy cows and provide theoretical support for precision management and milk quality control.

## 2. Materials and Methods

### 2.1. Animal Welfare Statement

The experiment was performed at the dairy farm of the Shandong Yinxiang Weiye Group Company (Cao County, Heze, China, 115°26′ E, 34°50′ N). The farm keeps Holstein cows for milk production. All experiments were approved by the Animal Ethics Committee of the Chinese Academy of Agricultural Sciences (Beijing, China, approval number IAS2023-68).

### 2.2. Animals, Diet, Management and Behavioral Observations

Based on the management practices of the study farm, all primiparous cows were housed in a single designated area comprising three large cowsheds, whereas multiparous cows were housed in separate cowsheds across different areas. Conducting behavioral observations on a large herd across these dispersed locations would have necessitated frequent movement by observers. Therefore, to exclude potential confounding effects associated with parity and to minimize observer workload, we selected primiparous cows as the subjects for this study.

A total of 916 healthy primiparous Holstein cows were housed in 3 large cowsheds. Each cowshed contained 2 pens, and each pen was equipped with 200 individual stalls. The cows were milked 3 times daily at 08:00, 15:00, and 20:00. After each milking session, cows were fed total mixed ration (TMR) at 08:30, 15:30, and 20:30. The detailed dietary composition is provided in Table A1. Farm veterinarians conducted daily health examinations and performed body condition score (BCS) monthly according to the methodology described by Edmondson et al. [32]. In the study farm, cows with extremely low (thin) or high (fat) BCS were routinely culled or managed separately according to standard farm management practices. As a result, the BCS of cows housed in the three observed cowsheds was relatively homogeneous and concentrated at 2.75, 3.00, and 3.25. Milk yield and days in milk (DIM) were automatically recorded daily by an automated milking system.

Three trained observers, who had participated in multiple dairy cow behavioral observation trials prior to this study and were capable of accurately assessing stereotypic behaviors, conducted behavioral observations on 916 cows over a 10-day period (21 to 30 April 2023) using the scan sampling behavior observation method. The stereotypic behaviors observed included TR, pica, feed-tossing, inter-sucking, head-shaking, excessive grooming, excessive vocalizing, and excessive rubbing. Detailed definitions of these behaviors are provided in Table A2. Prior to the formal observation period, a 3-day pre-observation trial was conducted. The prevalence-adjusted bias-adjusted kappa (PABAK) value ranged from 0.92 to 0.98 (mean = 0.94), indicating a high level of agreement among the 3 observers in identifying stereotypic behaviors. No cows were removed from the herd during the observation period. During each behavioral observation, the 3 observers were randomly assigned to the 3 cowsheds. Each observer was rotated to a different cowshed on the following day, so that every observer had the opportunity to assess all cowsheds within a 3-day cycle. In each behavior observation, the observer slowly walked from one end of the cowshed to the other, recording observations for all cows in that shed. Each cow was assessed only at the moment it was encountered, rather than being continuously observed, consistent with the definition of instantaneous scan sampling [7,8,33]. If a cow exhibited any stereotypic behaviors listed in Table A2 during a behavior observation, it was recorded as a single occurrence, along with the cow’s ID and the specific behavior type. It is important to note that repeated circular movements of the tongue occurring while the cow is feeding or ruminating is not classified as stereotypic TR. Each behavior observation lasted 10 min, with a 10 min interval between sessions. Although each observation session covered a 10 min period, individual cows were sampled instantaneously rather than continuously. Ten sessions were conducted in the morning (08:00–11:10) and ten in the afternoon (14:00–17:10) each day, resulting in 200 behavioral observation sessions over 10 days.

During animal experimentation stage of the behavioral observation and sample collection, ambient temperature (T) and relative humidity (RH) were recorded every 10 min using Kestrel 5400 Heat Stress Trackers (Nielsen-Kellerman, Boothwyn, PA, USA). The temperature–humidity index (THI) was calculated using the following equation: THI = (1.8 × T + 32) − (0.55 − 0.0055 × RH) × (1.8 × T − 26) [34]. Throughout the farm experimentation period, the THI in the cowsheds averaged 59.60 ± 6.62, showing the very suitable thermal environment.

### 2.3. Cow Grouping and Sample Collection

Prior to the start of the experiment, we planned to collect hair samples from 600 cows to assess their chronic stress levels based on HCC. In fact, 601 hair samples were successfully collected. Each morning before behavioral observation, 40 cows were randomly selected for hair sampling. Hair samples were collected from the tail switch area, cutting approximately 3 cm of hair closest to the skin. Sampling was conducted for one hour each day. After collecting the sample from each cow, the cow was marked with a red livestock crayon to avoid repeated sampling. The hair sampling process lasted 15 days; therefore, after the 10-day behavioral observation period, sampling continued for an additional 5 days. Following collection, HCC was measured using an enzyme-linked immunosorbent assay (ELISA), following the protocol described by Burnett et al. [35]. The assay kits were provided by Shanghai Hengyuan Biotechnology Co., Ltd. (Shanghai, China).

**Figure 1:**
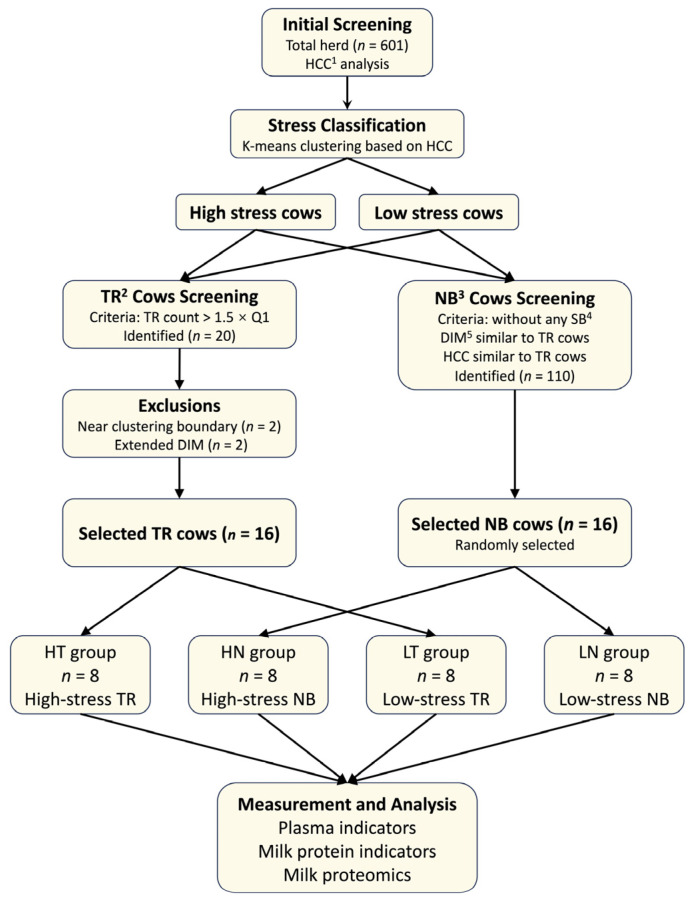
Cow grouping process and sample testing item. ^1^ HCC: hair cortisol concentration; ^2^ TR: tongue-rolling; ^3^ NB: normal behavior (i.e., no stereotypic behaviors); ^4^ SB: stereotypic behaviors; ^5^ DIM: days in milk.

First, the stress status of the entire herd (*n* = 601) was determined. Cows were classified into high-stress and low-stress populations using K-means clustering based on HCC values (Figure 1).

Following stress classification, behavioral screening was conducted to establish the study groups. Based on our previous research [2], cows whose observed TR counts exceeded 1.5 times the first quartile were identified as having high levels of TR, yielding 20 candidate cows.

To ensure clear separation of stress levels and to control for the effect of DIM, two cows located near the HCC clustering boundary and two cows with extended DIM were excluded. The remaining 16 cows were exactly assigned to the high-stress TR group (HT, *n* = 8; HCC = 16.01 ± 0.80 pg/mg, DIM = 116 ± 17) and the low-stress TR group (LT, *n* = 8; HCC = 11.14 ± 1.40 pg/mg, DIM = 131 ± 29), according to their initial stress classification.

To establish appropriate NB (without any stereotypic behaviors listed in Table A2) controls, cows with DIM and HCC values comparable to those of the high-stress and low-stress TR groups were first identified, resulting in a pool of 110 candidate cows. From this pool, 8 high-stress and 8 low-stress cows were randomly selected to form the high-stress NB group (HN, *n* = 8; HCC = 15.92 ± 0.44 pg/mg, DIM = 121 ± 30) and the low-stress NB group (LN, *n* = 8; HCC = 11.23 ± 0.89 pg/mg, DIM = 126 ± 33), respectively.

This sample size was validated by a power analysis using G*Power software (v3.1.9.6) and adhered to one of the 3Rs principle (Reduction) to minimize animal use. Hair cortisol concentration (HCC) was defined as the primary outcome variable. Post-hoc analysis confirmed that the sample size provided > 0.99 statistical power for the primary outcome (Cohen’s d = 4.27) and sufficient sensitivity (Power = 0.80) to detect effect sizes of d ≈ 1.51 in subsequent milk protein composition, plasma indicators, and milk proteome analysis.

For comparison, HT and LT cows were collectively referred to as TR cows, while HN and LN cows were referred to as NB cows; HT and HN cows were collectively referred to as high-stress cows, while LT and LN cows were collectively referred to as low-stress cows.

After grouping, the selected cows were examined by the farm veterinarian to ensure that all selected cows were in good health and free from any diseases. Subsequently, a 3-day behavioral observation was conducted on the selected cows to confirm that cows in the HT and LT groups continued to exhibit TR, while cows in the HN and LN groups did not show any stereotypic behaviors. Before the morning feeding on the first day following this 3-day observation, blood samples were collected from the coccygeal vein using a 20 mL syringe and divided into two heparin-coated tubes. All samples were centrifuged at 3000 rpm for 10 min at 4 °C. Plasma was then separated and aliquoted into four 2 mL cryovials, initially frozen in liquid nitrogen and later transferred to a −80 °C freezer for storage until analysis of immune and inflammatory indicators.

On the day following blood sampling, milk samples were collected from the selected cows. Samples were taken during the 08:00, 15:00, and 20:00 milking sessions in a 4:3:3 volume ratio and placed into 100 mL sterile plastic vials. Each sample was frozen in liquid nitrogen, then transferred to a −80 °C freezer for storage prior to milk protein and proteomic analysis.

### 2.4. Biochemical Testing of Plasma and Milk Sample

To evaluate milk protein composition, frozen milk samples were analyzed for αs1-CN, αs2-CN, β-CN, κ-CN, α-LA, β-LG, and LF. These components were quantified using ELISA-based enzyme immunoassays. The kits were provided by Shanghai Hengyuan Biotechnology Co., Ltd. (Shanghai, China).

To evaluate systemic immune function and inflammatory status, frozen plasma samples were analyzed for immunoglobulin A (IgA), immunoglobulin G (IgG), immunoglobulin M (IgM), lipopolysaccharide (LPS), tumor necrosis factor-α (TNF-α), interleukin-2 (IL-2), interleukin-6 (IL-6), and interleu-kin-10 (IL-10), which are widely used indicators to assess immune competence and inflammatory responses in dairy cows. IgA, IgG, IgM, and LPS were measured using colorimetric assays, while TNF-α, IL-2, IL-6, and IL-10 were determined using ELISA. All testing kits were supplied by the Beijing Sinouk Institute of Biological Technology (Beijing, China).

### 2.5. Data-Independent Acquisition Proteomics Analyses

Frozen milk samples were also used for proteomics analysis. Peptide fractions were prepared according to the filter-aided sample preparation protocol described by Wiśniewski et al. [36] and redissolved in 10 µL of 0.1% formic acid (FA) solution. Peptides were separated using a Vanquish ultra-high-performance liquid chromatography system (Thermo Fisher Scientific, Waltham, MA, USA) equipped with a nanoViper C18 column (75 µm × 250 mm, 2 µm; Thermo Fisher Scientific). The mobile phase A consisted of 0.1% FA in water (Sigma-Aldrich, St. Louis, MO, USA), and mobile phase B was composed of 80% acetonitrile with 0.1% FA. The separated peptides were then analyzed and identified using an Orbitrap Exploris 480 mass spectrometer (Thermo Fisher Scientific, Waltham, MA, USA).

### 2.6. Statistical Analysis of Plasma and Milk Indicators

To evaluate the effects of behavior (TR vs. NB), chronic stress (high vs. low), and their interaction effects on milk biochemical and plasma immune-inflammatory indicators, two-way ANOVA was performed using SPSS software version 26.0 (SPSS Inc., Chicago, IL, USA). BCS (fixed effect, BCS = 2.75, 3.00, 3.25) and DIM (continuous covariate) were also included to adjust for biological variations. All milk and plasma variables were checked for normality and homogeneity of variance and met the assumptions of ANOVA.

The complete statistical model can be expressed as:

*Y_ijkl_* = *μ* + *B_i_* + *S_j_* + (*B* × *S*)*_ij_* + *BCS_k_* + *β* (*DIM_ijkl_*) +*ε_ijkl_*

*Y_ijkl_* is the response variable (milk or plasma indicator), *μ* is the overall mean, *B_i_* is the fixed effect of behavior (*i* = TR, NB), *S_j_* is the fixed effect of chronic stress (*j* = high stress, low stress), *(B* × *S)_ij_* is the interaction between behavior and stress, *BCS_k_* is the fixed effect of body condition score, *β* is the regression coefficient associated with the continuous covariate DIM, *DIM_ijkl_* are covariates, and *ε_ijkl_* is the random residual error. Results are presented as mean ± SD, and differences were considered statistically significant at *p* < 0.05.

### 2.7. Statistical Analysis of Proteomics Data

Raw data files obtained from mass spectrometry were analyzed using DIA-NN version 1.8.1 (UK) with the UniProt Bos taurus (Bovine)_37503_20231123.fasta database to identify UniProt IDs for each protein.

We first assessed the differences in milk proteome profiles between TR cows and NB cows, as well as between high-stress cows and low-stress cows. Proteomics analysis was performed using the Limma package in R version 4.3.2. Differentially expressed proteins (DEPs) were identified based on the criteria of *p*-value < 0.05 and fold change (FC) > 1.2 or < 0.83, and were visualized using volcano plots. The UniProt IDs of DEPs were uploaded to the DAVID database [37] (https://davidbioinformatics.nih.gov, accessed on 15 July 2025, Frederick, MD, USA) for Kyoto Encyclopedia of Genes and Genomes (KEGG) pathway and Gene Ontology (GO) enrichment analyses, using Bos taurus as the reference species. KEGG pathway regulation direction (up- or downregulation) was assessed based on Z-score calculation. GO enrichment covered the categories of Biological Process (BP), Cellular Component (CC), and Molecular Function (MF). The UniProt IDs of DEPs were imported into the STRING [38] (https://string-db.org, accessed on 15 July 2025, Lausanne, Switzerland) for protein–protein interaction (PPI) analysis, with the minimum required interaction score set to 0.4.

For milk proteomic data, two-way ANOVA was performed using the same model as for milk and plasma indicators, with BCS and DIM included as fixed effect and continuous covariate, respectively. Proteomic data were first normalized and Log_10_-transformed to meet assumptions of normality and homogeneity of variance. Results are presented as mean ± SD, and differences were considered statistically significant at *p* < 0.05. Proteins with significant interaction effects (*p* < 0.05) were further subjected to GO and KEGG enrichment analyses using DAVID. Likely, the UniProt IDs of proteins with significant interaction effects were imported into the STRING for PPI analysis, using a minimum interaction score of 0.4.

Visualization of volcano plots and KEGG pathway enrichment results was performed using SRplot [39] (http://www.bioinformatics.com.cn, accessed on 15 July 2025, Shanghai, China), while GO enrichment plots were generated using GraphPad Prism 10 (Boston, MA, USA). The PPI network was visualized using Cytoscape 3.10.3 (San Diego, CA, USA).

## 3. Results

### 3.1. Plasma Immune-Inflammatory Indicators

Plasma immune-inflammatory indicators varied significantly with both TR and chronic stress level (Table 1). TR cows showed significantly lower IgA concentrations compared to NB cows (*p* < 0.05). Additionally, TR cows had significantly higher levels of LPS, TNF-α, and IL-6 (*p* < 0.05). High-stress cows exhibited significantly lower IgG concentrations than low-stress cows (*p* < 0.05), while IL-6 levels were significantly higher in the high-stress group (*p* < 0.05). A significant interaction effect between behavior (TR vs. NB) and chronic stress level was observed for TNF-α and IL-6 (*p* < 0.05). Specifically, HT cows exhibited the highest plasma concentrations of both TNF-α and IL-6, whereas the other three groups showed relatively similar and lower levels.

### 3.2. Milk Protein Indicators

Significant differences in milk protein components were observed between TR cows and NB cows (Table 2). Specifically, TR cows had significantly lower concentrations of LF, αs1-CN, β-CN, and κ-CN compared to NB cows (*p* < 0.05).

No significant main effect of stress level was detected for any of the milk protein indicators determined (*p* > 0.05), and no significant interaction effect between behavior (TR vs. NB) and chronic stress level was found (*p* > 0.05).

### 3.3. Milk Proteomics Results

**Figure 2:**
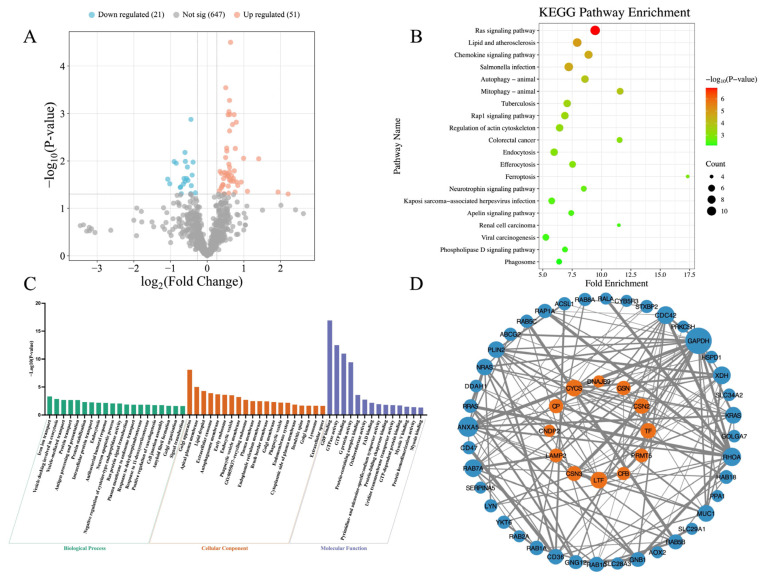
Differential proteomic profiles between tongue-rolling cows and normal behavior cows. (**A**) Volcano plot of differentially expressed proteins (DEPs). Each point represents a protein. Red and blue dots indicate significantly upregulated and downregulated DEPs in tongue-rolling cows, respectively, compared with normal behavior cows. A total of 51 proteins were significantly upregulated and 21 downregulated; (**B**) KEGG pathway enrichment analysis of DEPs. A total of 38 pathways were significantly enriched; the top 20 ranked by *p*-value are shown. Dot size indicates the number of proteins in each pathway, and color reflects −Log_10_(*p*-value), with red indicating a smaller *p*-value and green indicating a larger *p*-value; (**C**) GO enrichment analysis. Bars represent significantly enriched GO terms, including 27 biological processes (BP), 27 cellular components (CC), and 14 molecular functions (MF). The top 20 GO terms ranked by *p*-value are shown for BP and CC, while all enriched terms are displayed for MF. Bar height represents the −Log_10_(*p*-value) of the GO terms, with higher bars indicating smaller *p*-values. Bar colors: green (BP), orange (CC), purple (MF); (**D**) Protein–protein interaction (PPI) network. Each node represents a protein; blue indicates upregulation and orange indicates downregulation in TR cows. Node size reflects the number of interacting proteins—larger nodes represent proteins with more interactions. Lines connecting nodes represent interactions between proteins. The thickness of the line reflects the combined_score, with thicker lines indicating higher scores.

Figure 2 presents the differences in the milk proteomics between TR cows and NB cows. Compared to the NB cows, a total of 72 DEPs were identified in the TR cows, consisting of 51 significantly upregulated and 21 significantly downregulated proteins (Figure 2A). KEGG pathway enrichment analysis revealed 38 significantly enriched pathways. Notably, the majority of these pathways were upregulated in the TR cows, with only Ferroptosis (Z-score = −1.000, *p* = 0.001) and Mineral absorption (Z-score = −0.577, *p* = 0.033) being significantly downregulated (Figure 2B). Only the top 20 KEGG pathways ranked by *p*-value are shown in Figure 2B, while all enriched KEGG pathways are listed in Table A3. GO enrichment analysis identified 68 significantly enriched GO terms, including 27 terms under BP, 27 terms under CC, and 14 terms under MF. For BP and CC, only the top 20 GO terms ranked by *p*-value are shown in Figure 2C. A complete list of enriched GO terms is provided in Table A4. The PPI network is shown in Figure 2D. A total of 50 DEPs were involved in 136 interactions. GAPDH, ANXA5, RHOA, XDH, CDC42, and RAB7A were identified as central hub proteins in the network, each interacting with more than 10 other proteins.

**Figure 3:**
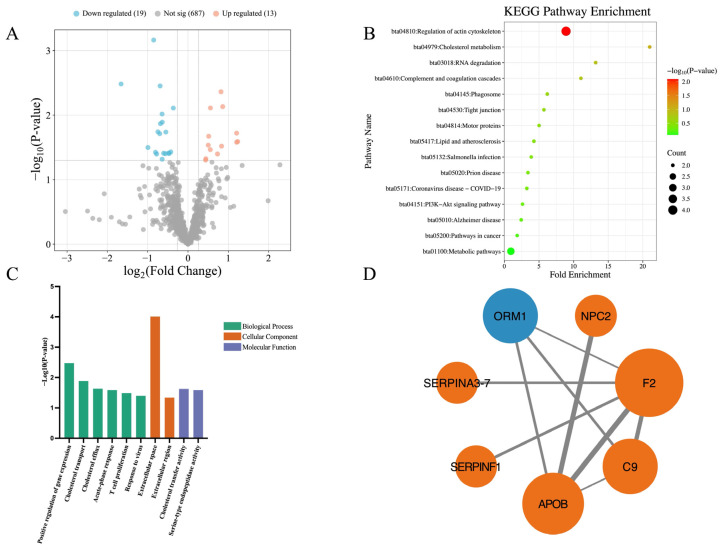
Differential proteomic profiles between high-stress cows and low-stress cows. (**A**) Volcano plot of differentially expressed proteins (DEPs). Each point represents a protein. Red and blue dots indicate significantly upregulated and downregulated DEPs in high-stress cows, respectively, compared with low-stress cows. A total of 13 proteins were significantly upregulated and 19 downregulated; (**B**) KEGG pathway enrichment analysis of DEPs. Only 1 pathway was significantly enriched (Regulation of actin cytoskeleton). Dot size indicates the number of proteins in each pathway, and color reflects −Log_10_(*p*-value), with red indicating a smaller *p*-value and green indicating a larger *p*-value; (**C**) GO enrichment analysis. Bars represent significantly enriched GO terms, including 6 biological processes (BP), 2 cellular components (CC), and 2 molecular functions (MF). Bar height represents the −Log_10_(*p*-value) of the GO terms, with higher bars indicating smaller *p*-values. Bar colors: green (BP), orange (CC), purple (MF); (**D**) Protein–protein interaction (PPI) network. Each node represents a protein; blue indicates upregulation and orange indicates downregulation in high-stress cows. Node size reflects the number of interacting proteins—larger nodes represent proteins with more interactions. Lines connecting nodes represent interactions between proteins. The thickness of the line reflects the combined_score, with thicker lines indicating higher scores.

Figure 3 illustrates the differences in the milk proteomics between high-stress and low-stress cows. Compared to the low-stress cows, a total of 32 DEPs were identified in the high-stress cows, consisting of 13 significantly upregulated and 19 significantly downregulated proteins (Figure 3A). KEGG pathway enrichment analysis revealed only 1 significantly enriched pathway, which was downregulated in high-stress cows: Regulation of actin cytoskeleton (Z-score = −2.000, *p* = 0.008) (Figure 3B). GO enrichment analysis identified 10 significantly enriched GO terms, including 6 terms under BP, 2 under CC, and 2 under MF (Figure 3C). The PPI network included only 7 DEPs, forming 9 interaction relationships (Figure 3D).

**Figure 4:**
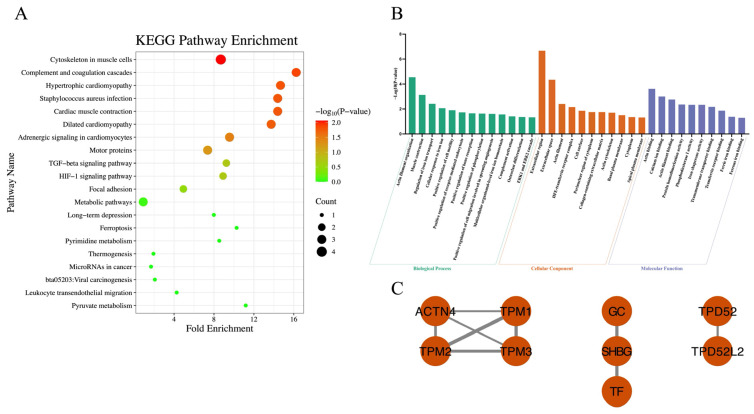
Proteins in the milk proteome with significant interaction effects between behavior (tongue-rolling vs. normal behavior) and chronic stress. (**A**) KEGG pathway enrichment analysis of 28 proteins. A total of 7 pathways were significantly enriched; the top 20 ranked by *p*-value are shown; (**B**) GO enrichment analysis of the 28 proteins. A total of 34 GO terms were significantly enriched, including 13 biological process (BP), 11 cellular components (CC) and 10 molecular functions (MF). Bar colors: green (BP), orange (CC), purple (MF); (**C**) The protein–protein interaction of 9 proteins. Each node represents a protein, and edges represent interactions between proteins. The thickness of each edge indicates the combined_score, with thicker edges representing stronger interactions.

A two-way ANOVA was performed on the proteomic data to examine the main and interaction effects of behavior (TR vs. NB) and chronic stress level. As shown in Table 3, a significant interaction between behavior (TR vs. NB) and chronic stress levels was identified for 28 proteins. KEGG pathway enrichment analysis revealed 7 significantly enriched pathways (Figure 4A). GO enrichment analysis revealed 34 significantly enriched GO terms, including 13 under BP, 11 under CC and 10 under MF (Figure 4B). PPI analysis revealed that the proteins were relatively scattered and did not form an integrated network (Figure 4C).

## 4. Discussion

### 4.1. Immune Function and Inflammatory Responses

During lactation, dairy cows exhibit high metabolic activity and energy demand, and are frequently exposed to various stressors such as overcrowding, poor ventilation, and routine veterinary procedures. These adverse factors not only impair milk production and animal welfare but also affect immune function and anti-inflammatory capacity, thereby increasing disease susceptibility [40,41,42]. IgA, IgG, and IgM, mainly secreted by B lymphocytes, are the key components of humoral immunity. IgA is the most abundant antibody at mucosal surfaces and serves as the first line of defense against pathogens [43,44]. Moreover, IgA has been suggested as a potential indicator of animal welfare. Previous studies reported that serum IgA levels were significantly lower in high-anxiety mice [45], in horses subjected to intense sanitary and training regimens [46], and in broilers raised under high stocking density [43]. These findings indicate that reduced welfare or negative environmental factors can significantly suppress IgA levels and impair mucosal immune function. In our study, TR cows showed significantly lower plasma IgA levels compared to NB cows, suggesting a potential decline in mucosal function. Given that stereotypic behaviors are generally considered indicators of poor welfare, our findings align well with previous studies, suggesting that tongue-rolling cows may exhibit physiological manifestations of decreased immune capacity [3]. It is noteworthy that plasma IgM levels showed no significant differences between tongue-rolling and non-tongue-rolling cows, nor between high- and low-stress groups. This stability may be attributed to the role of IgM as a primary systemic defense antibody, which tends to maintain relatively stable levels within the bovine organism [47]. However, no significant interaction effect between behavior (TR vs. NB) and chronic stress level was observed for IgA levels.

LPS, also known as endotoxin, is a structural component of the outer membrane of Gram-negative bacteria. In dairy cows, high-concentrate feeding can disrupt digestive homeostasis and induce subacute ruminal acidosis (SARA), which negatively affects production performance [40]. Increasing evidence suggests that SARA promotes the growth and lysis of Gram-negative bacteria, leading to the release of large amounts of LPS. LPS can translocate from the gastrointestinal tract into the bloodstream and mammary gland, where it activates the LPS/TLR4 signaling pathway, triggering inflammatory responses and promoting the release of proinflammatory cytokines such as IL-1β, IL-6, and TNF-α [48]. IL-1β can further activate STAT3 and NF-κB signaling to induce the expression of IL-6 and TNF-α, amplifying the inflammatory cascade [49]. In our study, TR cows showed significantly higher plasma levels of LPS, IL-6, and TNF-α compared to NB cows, indicating a heightened systemic inflammatory response. The elevated TNF-α levels may also contribute to the observed reduction in β-CN concentration in milk from TR cows. Furthermore, we observed significant interaction effects between behavior (TR vs. NB) and chronic stress levels on plasma IL-6 and TNF-α concentrations. In TR cows, high-stress further exacerbated the inflammatory response. TR has been classified as a non-nutritive oral behavior, and one of its causes is excessive intake of concentrate feed [6]. Sun et al. [7] found that cows with TR had significantly lower rumen pH than cows with normal behavior, supporting the hypothesis of subclinical rumen dysfunction. The alkalinity of saliva helps to buffer rumen acidity during rumination [2,50]. The expression of TR may indicate insufficient physical structure in the ration. Inadequate dietary structure can reduce mastication activity and saliva secretion, thereby compromising the ruminal buffering capacity and increasing susceptibility to ruminal acidosis. In our study, the elevated plasma LPS levels in TR cows may reflect increased intestinal translocation due to dietary factors, ultimately contributing to reduced milk protein quality.

In summary, compared to NB cows, TR cows exhibited impaired immune function and a heightened inflammatory response. Moreover, when exposed to high-stress, TR cows showed an exacerbation of systemic inflammation.

### 4.2. Milk Protein Quality

Milk proteins are rich in essential amino acids and highly digestible, making them the optimal source of protein for neonatal calves [51]. The synthesis of milk proteins requires adequate dietary energy and crude protein intake [52]. Milk proteins are mainly composed of caseins and whey proteins, with caseins accounting for approximately 80% of total milk proteins [53,54]. Their synthesis is highly dependent on the uptake of amino acids by mammary epithelial cells, particularly essential amino acids such as methionine and lysine [55,56]. Caseins include four major types: αs1-CN, αs2-CN, β-CN, and κ-CN [57]. In our previous study, we found that cows exhibiting high levels of TR produced milk with significantly lower percentages of total milk protein, crude protein, and true protein compared to normal behavior cows [2]. Similar findings were observed in the current study, where concentrations of αs1-CN, β-CN, and κ-CN were significantly lower in the milk of TR cows. Tsugami et al. [58] reported that pro-inflammatory cytokines such as IL-1β and TNF-α inhibit β-CN secretion, thereby impairing milk protein synthesis. These findings collectively indicate that TR may be associated with reduced milk protein content and compromised milk quality. In contrast, regarding the two whey proteins, α-LA and β-LG, no significant differences were observed between TR and NB cows. It has been reported that the levels of α-LA and β-LG remain unchanged in diseased cows [59,60]. This suggests that the synthesis of α-LA and β-LG in the mammary gland is relatively stable; therefore, alterations in whey proteins may not be as pronounced as those observed in caseins.

Physiological stress negatively affects the productivity of livestock, including reductions in milk yield and milk protein production in dairy cows. Dado-Senn et al. [61] reported that cows exposed to heat stress during the dry period exhibited a 0.18 kg/day reduction in milk protein yield during the subsequent lactation. Similarly, exposure to heat stress during lactation has been shown to decrease both total milk protein and casein yields [62]. Transport-induced stress has also been associated with significant declines in milk yield and quality [63]. However, in the present study, cows with different levels of chronic stress showed no significant differences in milk protein indicators. This may be attributed to the fact that the stress levels were based on inherent individual variations rather than externally imposed stressors. While the hair cortisol levels effectively distinguished the groups, these internal physiological differences may stay within a range that the mammary gland can compensate for. This suggests that milk protein synthesis is relatively stable under inherent physiological state of high cortisol level, and more intense or acute internal stressors (such as clinical disease or systemic inflammation) or severe external stressors (such as extreme heat stress or physical trauma) might be required to cause significant changes in milk protein fractions.

### 4.3. Milk Proteomics

Proteomics is a powerful technique commonly used to investigate the composition and dynamic changes in proteins in animal tissues and body fluids [64]. In recent years, proteomic approaches have been widely applied in ruminant studies, including research on animal nutrition [65], genetics [66], and ruminant disease mechanisms [67].

Proteomics revealed substantial differences between TR cows and NB cows, with many of the DEPs belonging to the Ras superfamily. Ras proteins are core components of guanosine triphosphate (GTP)-binding proteins and are classified into five major subfamilies: Rab, Rho, Ras, Rap, and Ran [68,69]. Rab proteins are key regulators of intracellular vesicle trafficking, coordinating membrane transport and organelle interactions critical for maintaining cellular metabolism and homeostasis [70,71]. Rho family GTPases (e.g., RhoA, CDC42) modulate cytoskeletal organization, cell polarity, and migration through pathways like mDia and ROCK [72]. Additionally, Ras and Rap proteins, though structurally related, often exert opposing effects on cell signaling—Ras proteins promote growth and differentiation, while Rap proteins can suppress excitatory signaling [73,74]. In this study, several Ras superfamily proteins, including Rab1A, Rab5B, Rab10, RhoA, RRas, and Rap1A, were significantly upregulated in the milk of TR cows compared to NB cows, suggesting enhanced metabolic activity and altered vesicular transport processes.

In this study, proteomics differences between high-stress and low-stress cows were relatively limited. Among these, only one KEGG pathway—regulation of actin cytoskeleton—was significantly enriched and found to be downregulated. The actin cytoskeleton is one of the most essential intracellular structural frameworks, playing critical roles in maintaining cell morphology, facilitating cellular movement, and regulating cell division. 4 DEPs were involved in this pathway: C9, Moesin, G protein subunit gamma 12, and Thrombin. The downregulation of this pathway may reflect subtle changes in cytoskeletal organization or intracellular signaling in response to chronic stress. C9 is a component of the complement system and plays a key role in innate immune defense against pathogen invasion. In vertebrates, C9 and perforin form oligomeric pores that lyse bacteria and destroy virus-infected cells [75,76]. Moesin, originally isolated from bovine uterus, is a member of the ERM (Ezrin, Radixin, Moesin) protein family. As a cytoskeletal linker protein, it plays a critical role in cell movement and migration, and is also involved in wound healing processes [77,78]. G proteins are heterotrimeric signaling proteins composed of α, β, and γ subunits [79]. G protein subunit gamma 12 (GNG12) belongs to the Gγ family and is involved in regulating cell division, differentiation, and metastasis [80,81]. GNG12 has also been implicated in modulating immune responses. For instance, BV-2 microglial cells, which serve as immunoprotective cells in the nervous system, exhibit increased TNF-α expression when GNG12 is knocked down [82], indicating that GNG12 may act as an anti-inflammatory regulatory factor [83]. Thrombin is a serine protease that plays a central role in coagulation and is also involved in diverse biological processes such as cell differentiation and tissue remodeling [84,85]. In this study, all four of these proteins—C9, Moesin, GNG12, and Thrombin—were significantly downregulated in the milk of high-stress cows compared to low-stress cows. These findings suggest that chronic stress may compromise the cows’ antimicrobial and anti-inflammatory capacities, potentially increasing susceptibility to infection and impairing immune homeostasis.

A significant interaction between behavior (TR vs. NB) and chronic stress level was detected in the milk proteome. Among the proteins associated with this interaction, particular attention was given to iron-binding proteins. In ruminant milk, two major iron-binding proteins have been identified: LF and transferrin (TF). Transferrin, primarily synthesized in the liver, plays a central role in transporting Fe^3+^ and maintaining systemic iron homeostasis [86]. In dairy cows, TF in milk originates not only from plasma but can also be synthesized locally by mammary epithelial cells. Lee et al. [87] demonstrated that mammary epithelial cells in virgin female mice produce small amounts of TF, with levels increasing during pregnancy and lactation. Furthermore, TF levels in adipose tissue have been shown to increase under stress conditions such as oxidative stress or disease [88]. In the present study, milk from HN cows exhibited significantly elevated TF concentrations, consistent with a stress-induced regulatory response. However, this increase was not observed in HT cows, whose TF levels were lower than those of low-stress cows. This interaction pattern suggests that tongue-rolling behavior may disrupt the local regulation of TF expression in the mammary gland, thereby modulating its stress responsiveness. Although the precise mechanism remains to be elucidated, we hypothesize that the physical exertion and metabolic costs of TR behavior, combined with the associated inflammatory response (as indicated by elevated LPS), might interfere with iron-related protein synthesis. In addition, milk from TR cows exhibited significantly lower LF concentrations than that from NB cows. Given that both TF and LF are iron-binding proteins involved in local iron metabolism within the mammary gland, these findings suggest that TR may be associated with impaired iron-binding capacity in milk when cows are under chronic stress conditions.

Complement Factor H (CFH) is a key regulatory protein of the complement system, widely involved in both innate and adaptive immunity, and plays a role in tissue regeneration [89,90]. In the bovine mammary gland, the complement system is an essential component of the immune defense against infectious diseases such as mastitis. Rainard [91] reported that CFH, in cooperation with Factor I, cleaves complement component C3b into C3bi, which can then bind to the CR3 receptor on neutrophils. This process enhances neutrophil-mediated recognition and phagocytosis of pathogens, representing a crucial step in complement-mediated opsonization. In this study, we observed a significant increase in milk CFH levels in HN cows. However, this elevation was not detected in HT cows. This interaction pattern suggests that TR may be speculated to interfere with stress-induced upregulation of CFH expression in the mammary gland.

Despite the novel insights provided by this study, several limitations should be acknowledged. First, while the sample size was sufficient for proteomic analysis, it is relatively limited for capturing the full spectrum of behavioral heterogeneity; thus, larger cohorts are needed to validate these interaction effects. Secondly, the experiment was conducted exclusively on primiparous cows to control for physiological variations related to parity. Consequently, the results may not be directly extrapolatable to multiparous cows, which may exhibit different physiological responses to stress and stereotypies. Lastly, as a cross-sectional study, our findings highlight the interaction effects between tongue-rolling and chronic stress on milk proteome, but do not establish a definitive causal relationship. Whether the observed proteomic alterations trigger the behavioral phenotype or result from it remains to be elucidated. Future longitudinal research with larger sample sizes, involving more factors (such as parity, season, etc.) is warranted to further verify the molecular mechanisms underlying the interaction between stereotyped behaviors and stress physiology in dairy cows.

## 5. Conclusions

This study used a two-factor design to investigate the interaction between behavior (TR vs. NB) and chronic stress levels in dairy cows, focusing on milk proteomics, milk protein and plasma immune-inflammatory indicators. Significant interaction effects were observed for inflammatory cytokines (TNF-α, IL-6) and 28 milk proteins. Compared to NB cows, TR cows produced milk of lower quality, primarily characterized by reduced levels of LF, αs1-CN, β-CN, and κ-CN. Furthermore, TR cows exhibited decreased plasma IgA levels alongside increased levels of LPS, TNF-α, and IL-6, indicating compromised immunity and increased inflammation. These responses were particularly pronounced under high-stress physiological state, as reflected by the elevated TNF-α and IL-6 concentrations. Proteomic analysis further revealed that iron-binding proteins (e.g., TF) and complement regulators (e.g., CFH) were highly responsive to the behavior (TR vs. NB)–chronic stress interaction, implying altered local iron metabolism and immune defense. The findings of this study have practical implications for dairy farming. By identifying behavioral indicators such as tongue-rolling in combination with physiological measures of stress, farmers can better monitor individual cow welfare and implement targeted interventions. This approach can support precision management by enabling early detection of cows at risk of stress-related health or production issues. Furthermore, characterizing the associations between stereotypic behaviors, stress, and milk protein composition provides valuable insights for milk quality control, allowing producers to optimize herd management practices and maintain high-quality milk production.

## Figures and Tables

**Table 1:** Differences in plasma immune-inflammatory indicators between tongue-rolling and normal behavior cows under different stress levels (high vs. low).

Items	B ^1^	Level of Cortisol		*p*-Value		
		High	Low	B	LOC ^2^	B × LOC
IgA (g/L)	TR ^3^	2.60 ± 0.26	2.80 ± 0.26	**0.002**	0.218	0.494
	NB ^4^	3.01 ± 0.27	3.07 ± 0.34			
IgG (g/L)	TR	9.68 ± 0.64	10.17 ± 0.60	0.146	**0.023**	0.785
	NB	9.96 ± 0.63	10.57 ± 0.65			
IgM (g/L)	TR	0.70 ± 0.05	0.71 ± 0.04	0.184	0.845	0.449
	NB	0.69 ± 0.07	0.67 ± 0.08			
LPS (EU/mL)	TR	0.33 ± 0.02	0.32 ± 0.03	**0.002**	0.074	0.633
	NB	0.30 ± 0.03	0.28 ± 0.03			
TNF-α (pg/mL)	TR	52.85 ± 2.77	47.94 ± 2.67	**0.003**	0.078	**0.046**
	NB	46.28 ± 3.21	46.57 ± 4.74			
IL-2 (pg/mL)	TR	209.01 ± 32.90	221.54 ± 32.23	0.559	0.989	0.269
	NB	215.07 ± 33.90	202.22 ± 28.00			
IL-6 (pg/mL)	TR	128.07 ± 11.93	104.38 ± 9.36	**0.029**	**0.006**	**0.002**
	NB	107.07 ± 12.46	108.66 ± 7.78			
IL-10 (pg/mL)	TR	14.49 ± 0.88	14.52 ± 1.90	0.110	0.564	0.586
	NB	13.14 ± 1.51	13.84 ± 2.31			

^1^ B: behavior. ^2^ LOC: level of cortisol. ^3^ TR: the cows with tongue-rolling behavior. ^4^ NB: the cows with normal behavior. IgA: immunoglobulin A; IgG: immunoglobulin G; IgM: immunoglobulin M; LPS: lipopolysaccharide; TNF-α: tumor necrosis factor-α; IL-2: interleukin-2; IL-6: interleukin-6; IL-10: interleukin-10. Results are presented as mean ± SD. Significant values are highlighted in bold (*p* < 0.05).

**Table 2:** Differences in milk protein indicators and milk yield between tongue-rolling and normal behavior cows under different stress levels (high vs. low).

Items	B ^1^	Level of Cortisol		*p*-Value		
		High	Low	B	LOC ^2^	B × LOC
αs1-CN (μg/mL)	TR ^3^	55.29 ± 1.84	54.52 ± 0.99	**0.019**	0.348	0.638
	NB ^4^	56.38 ± 1.49	56.12 ± 1.59			
αs2-CN (μg/mL)	TR	40.70 ± 1.51	40.38 ± 1.91	0.394	0.495	0.816
	NB	41.45 ± 1.71	40.81 ± 2.45			
β-CN (μg/mL)	TR	98.53 ± 2.23	100.10 ± 2.53	**<0.001**	0.983	0.091
	NB	104.54 ± 2.50	102.93 ± 2.81			
κ-CN (μg/mL)	TR	23.90 ± 0.77	23.03 ± 0.77	**0.016**	0.059	0.403
	NB	24.42 ± 0.92	24.07 ± 0.93			
α-LA (μg/mL)	TR	26.07 ± 0.98	26.12 ± 1.19	0.295	0.548	0.648
	NB	26.26 ± 0.70	26.61 ± 0.53			
β-LG (μg/mL)	TR	337.05 ± 13.39	331.08 ± 9.46	0.068	0.959	0.108
	NB	337.91 ± 10.00	344.27 ± 7.59			
LF (μg/mL)	TR	783.90 ± 37.38	795.15 ± 26.39	**0.003**	0.319	0.989
	NB	820.50 ± 34.96	833.05 ± 31.87			
Milk yield (kg/d)	TR	33.67 ± 10.96	33.50 ± 7.51	0.454	0.325	0.589
	NB	33.46 ± 9.40	28.97 ± 8.19			

^1^ B: behavior. ^2^ LOC: level of cortisol. ^3^ TR: the cows with tongue-rolling behavior. ^4^ NB: the cows with normal behavior. αs1-CN: αs1-casein; αs2-CN: αs2-casein; β-CN: β-casein; κ-CN: κ-casein; α-LA: α-lactalbumin; β-LG: β-lactoglobulin; LF: lactoferrin. Results are presented as mean ± SD. Significant values are highlighted in bold (*p* < 0.05).

**Table 3:** Proteins in the milk proteome with significant interaction effects between behavior (tongue-rolling vs. normal behavior) and chronic stress levels (high vs. low).

Items	B ^1^	Level of Cortisol		*p*-Value		
		High	Low	B	LOC ^2^	B × LOC
TD52	TR ^3^	4.72 ± 0.14	4.88 ± 0.08	0.355	0.941	**0.034**
	NB ^4^	4.79 ± 0.17	4.60 ± 0.30			
AHNAK	TR	5.79 ± 0.26	5.78 ± 0.17	**0.002**	**0.009**	**0.025**
	NB	6.21 ± 0.13	5.88 ± 0.19			
ACTN4	TR	5.03 ± 0.37	4.78 ± 0.15	0.307	0.527	**0.048**
	NB	4.86 ± 0.27	5.22 ± 0.38			
SHBG	TR	5.15 ± 0.09	5.33 ± 0.10	0.574	0.601	**0.007**
	NB	5.36 ± 0.19	5.12 ± 0.19			
ICOSLG	TR	4.91 ± 0.18	5.05 ± 0.25	0.970	0.958	**0.050**
	NB	5.09 ± 0.17	4.92 ± 0.12			
GC	TR	6.28 ± 0.22	6.32 ± 0.10	0.105	**0.042**	**0.044**
	NB	6.59 ± 0.21	6.21 ± 0.27			
NUCB2	TR	6.57 ± 0.44	6.87 ± 0.12	0.113	0.213	**0.031**
	NB	6.71 ± 0.14	6.63 ± 0.15			
GPC1	TR	4.96 ± 0.23	4.93 ± 0.10	0.106	0.219	**0.044**
	NB	4.92 ± 0.23	5.17 ± 0.08			
KRT19	TR	5.50 ± 0.14	5.50 ± 0.15	0.137	**0.040**	**0.041**
	NB	5.35 ± 0.17	5.84 ± 0.36			
ENPP3	TR	6.07 ± 0.18	6.22 ± 0.08	0.066	0.595	**0.036**
	NB	6.09 ± 0.17	5.98 ± 0.17			
CPQ	TR	5.30 ± 0.18	5.12 ± 0.11	0.318	0.653	**0.006**
	NB	5.07 ± 0.14	5.26 ± 0.08			
CFH	TR	5.96 ± 0.14	6.01 ± 0.14	0.082	0.070	**0.008**
	NB	6.19 ± 0.09	5.97 ± 0.08			
TF	TR	7.00 ± 0.18	7.24 ± 0.15	0.008	0.893	**0.001**
	NB	7.38 ± 0.12	7.22 ± 0.08			
TM4SF18	TR	5.83 ± 0.13	5.80 ± 0.16	0.523	**0.026**	**0.047**
	NB	5.96 ± 0.22	5.67 ± 0.17			
FGFBP1	TR	5.60 ± 0.37	5.97 ± 0.08	0.385	0.813	**0.027**
	NB	5.97 ± 0.23	5.78 ± 0.28			
FST	TR	5.45 ± 0.28	4.92 ± 0.14	0.862	0.098	**0.024**
	NB	5.23 ± 0.30	5.33 ± 0.24			
TPM1	TR	5.25 ± 0.26	4.86 ± 0.09	0.740	0.813	**0.006**
	NB	4.94 ± 0.22	5.24 ± 0.30			
TGFBR3	TR	5.23 ± 0.34	5.07 ± 0.15	0.084	0.894	**0.030**
	NB	5.25 ± 0.16	5.40 ± 0.12			
TPD52L2	TR	4.49 ± 0.22	4.70 ± 0.08	0.751	0.250	**0.015**
	NB	4.67 ± 0.06	4.60 ± 0.08			
ENPP2	TR	4.78 ± 0.29	4.60 ± 0.18	0.584	0.881	**0.033**
	NB	4.55 ± 0.12	4.77 ± 0.08			
PRKG2	TR	4.88 ± 0.13	4.96 ± 0.07	0.333	0.399	**0.028**
	NB	5.03 ± 0.08	4.89 ± 0.12			
MASP1	TR	5.57 ± 0.24	5.23 ± 0.25	0.767	0.924	**0.035**
	NB	5.29 ± 0.25	5.58 ± 0.29			
LDHA	TR	5.05 ± 0.10	4.92 ± 0.19	0.164	0.096	**0.012**
	NB	4.81 ± 0.33	5.26 ± 0.15			
SARAF	TR	4.74 ± 0.15	4.46 ± 0.15	0.346	0.662	**0.046**
	NB	4.51 ± 0.41	4.79 ± 0.11			
THBS4	TR	4.83 ± 0.13	4.60 ± 0.20	0.202	0.143	**0.025**
	NB	4.63 ± 0.12	4.70 ± 0.07			
FIS1	TR	5.07 ± 0.11	4.86 ± 0.12	0.284	0.824	**0.025**
	NB	4.98 ± 0.09	5.09 ± 0.21			
TPM3	TR	4.81 ± 0.26	4.33 ± 0.25	0.245	0.357	**0.050**
	NB	4.52 ± 0.17	4.97 ± 0.55			
TPM2	TR	4.80 ± 0.29	4.44 ± 0.23	0.536	0.910	**0.012**
	NB	4.56 ± 0.26	4.85 ± 0.31			

^1^ B: behavior. ^2^ LOC: level of cortisol. ^3^ TR: the cows with tongue-rolling behavior. ^4^ NB: the cows with normal behavior. Results are presented as mean ± SD. TPD52: Tumor protein D52; AHNAK: AHNAK nucleoprotein; ACTN4: Alpha-actinin-4; SHBG: Sex hormone-binding globulin; ICOSLG: ICOS ligand; GC: Vitamin D-binding protein; NUCB2: Nucleobindin 2; GPC1: Glypican-1; KRT19: Keratin, type I cytoskeletal 19; ENPP3: Ectonucleotide pyrophosphatase family member 3; CPQ: Carboxypeptidase Q; CFH: Complement factor H; TF: Transferrin; TM4SF18: Transmembrane 4 L6 family member 18; FGFBP1: Fibroblast growth factor-binding protein 1; FST: Follistatin; TPM1: Tropomyosin alpha-1 chain; TGFBR3: Transforming growth factor beta receptor type 3; TPD52L2: TPD52 like 2; ENPP2: Autotaxin; PRKG2: cGMP-dependent protein kinase; MASP1: Complement C1s subcomponent; LDHA: L-lactate dehydrogenase A chain; SARAF: Store-operated calcium entry-associated regulatory factor; THBS4: Thrombospondin-4; FIS1: Mitochondrial fission 1 protein; TPM3: Tropomyosin alpha-3 chain; TPM2: Tropomyosin beta chain. Significant values are highlighted in bold (*p* < 0.05).

## Data Availability

The original contributions presented in this study are included in the article. Further inquiries can be directed to the corresponding author.

## Abbreviations

The following abbreviations are used in this manuscript:

ACTN4	Alpha-actinin-4
AHNAK	AHNAK nucleoprotein
BCS	Body condition score
BP	Biological process
CC	Cellular component
CFH	Complement factor H
CPQ	Carboxypeptidase Q
DEP	Differentially expressed protein
DIM	Days in milk
ELISA	Enzyme-linked immunosorbent assay
ENPP2	Autotaxin
ENPP3	Ectonucleotide pyrophosphatase family member 3
FA	Formic acid
FC	Fold change
FGFBP1	Fibroblast growth factor-binding protein 1
FIS1	Mitochondrial fission 1 protein
FST	Follistatin
GC	Vitamin D-binding protein
GNG12	G protein subunit gamma 12
GO	Gene ontology
GPC1	Glypican-1
GTP	Guanosine triphosphate
HCC	Hair cortisol concentration
HN	High-stress normal behavior cows
HPA	Hypothalamic–pituitary–adrenal
HT	High-stress tongue-rolling cows
ICOSLG	ICOS ligand
IgA	Immunoglobulin A
IgG	Immunoglobulin G
IgM	Immunoglobulin M
IL-10	Interleukin-10
IL-2	Interleukin-2
IL-6	Interleukin-6
KEGG	Kyoto Encyclopedia of Genes and Genomes
KRT19	Keratin, type I cytoskeletal 19
LDHA	L-lactate dehydrogenase A chain
LF	Lactoferrin
LN	Low-stress normal behavior cows
LOC	Level of cortisol
LPS	Lipopolysaccharide
LT	Low-stress tongue-rolling cows
MASP1	Complement C1s subcomponent
MF	Molecular function
NB	Normal behavior
NUCB2	Nucleobindin 2
PABAK	Prevalence-adjusted bias-adjusted kappa
PPI	Protein–protein interaction
PRKG2	cGMP-dependent protein kinase
RH	Relative humidity
SARA	Subacute ruminal acidosis
SARAF	Store-operated calcium entry-associated regulatory factor
SHBG	Sex hormone-binding globulin
T	Temperature
TF	Transferrin
TGFBR3	Transforming growth factor beta receptor type 3
THBS4	Thrombospondin-4
THI	Temperature–humidity index
TM4SF18	Transmembrane 4 L6 family member 18
TMR	Total Mixed Ration
TNF-α	Tumor necrosis factor-α
TPD52	Tumor protein D52
TPD52L2	TPD52 like 2
TPM1	Tropomyosin alpha-1 chain
TPM2	Tropomyosin beta chain
TPM3	Tropomyosin alpha-3 chain
TR	Tongue-rolling
α-LA	α-lactalbumin
αs1-CN	αs1-casein
αs2-CN	αs2-casein
β-CN	β-casein
β-LG	β-lactoglobulin
κ-CN	κ-casein

## Appendix A

**Table A1:** Ingredients and nutrient composition of experimental diet (%, dry matter basis).

Items	Value
**Ingredients**	**Content, %**
Alfalfa	10.39
Oat hay	2.42
Dandelion	0.48
Whole corn silage	48.33
Cottonseed	2.90
Beet pulp	2.42
Ground corn	7.49
Pressed corn	9.42
Soybean meal	8.70
Rapeseed meal	1.69
DDGS ^1^	0.72
Extruded soybean	1.33
Mineral and vitamin mix ^2^	3.70
**Nutrient composition**	
DM, % of wet TMR	62.40
CP	17.06
EE	3.32
NDF	35.75
ADF	18.20
NEL/(MJ/kg)	6.11

^1^ DDGS, Distillers Dried Grains with Solubles; ^2^ Contained the following per kg of diets: VA 170,000 IU, VD 8000 IU, VE 9000 IU, Ca 160 g, Fe 800 mg, Cu 680 mg, Mn 3500 mg, Zn 7500 mg, Se 80 mg, I 400 mg, Co 38 mg.

**Table A2:** Descriptions of the stereotypic behaviors [2].

Behaviors	Descriptions
Tongue-rolling	The cow’s tongue makes repeated circular movements inside and outside the mouth while neither eating nor ruminating.
Pica	The cow licks or bites non-food objects (e.g., fences, food trough).
Feed-tossing	The cow picks up a mouthful of feed and throws it into the air.
Inter-sucking	The cow sucks on its own or other cows’ body parts.
Head-shaking	The cow makes its head move quickly from side to side.
Excessive-grooming	The cow grooms itself in the same area for more than 10 s.
Excessive-vocalizing	The cow keeps vocalizing for more than 10 s.
Excessive-rubbing	The cow chafes its head or parts of its body against cowshed structures or equipment for more than 10 s.

**Table A3:** Kyoto Encyclopedia of Genes and Genomes (KEGG) pathway of differential proteins between tongue-rolling and normal behavior cows.

Pathway Name	*p*-Value	Fold Enrichment	Z-Score
Ras signaling pathway	<0.001	9.462	3.317
Lipid and atherosclerosis	<0.001	7.934	2.333
Chemokine signaling pathway	<0.001	8.899	2.828
Salmonella infection	<0.001	7.216	2.333
Autophagy-animal	<0.001	8.597	1.890
Mitophagy-animal	<0.001	11.589	2.449
Tuberculosis	<0.001	7.082	1.134
Rap1 signaling pathway	<0.001	6.885	2.646
Regulation of actin cytoskeleton	<0.001	6.438	1.890
Colorectal cancer	<0.001	11.547	1.342
Endocytosis	<0.001	5.973	2.646
Efferocytosis	0.001	7.543	2.449
Ferroptosis	0.001	17.343	−1.000
Neurotrophin signaling pathway	0.003	8.498	2.236
Kaposi sarcoma-associated herpesvirus infection	0.003	5.768	1.633
Apelin signaling pathway	0.004	7.429	2.236
Renal cell carcinoma	0.005	11.484	2.000
Viral carcinogenesis	0.005	5.268	1.633
Phospholipase D signaling pathway	0.007	6.898	2.236
Phagosome	0.009	6.399	1.342
Axon guidance	0.013	5.869	2.236
MAPK signaling pathway	0.012	4.249	2.449
C-type lectin receptor signaling pathway	0.013	8.017	2.000
Pathways in cancer	0.013	3.057	2.121
Proteoglycans in cancer	0.014	5.182	2.236
Cholinergic synapse	0.016	7.390	2.000
Serotonergic synapse	0.018	6.966	2.000
AMPK signaling pathway	0.020	6.745	2.000
T cell receptor signaling pathway	0.021	6.639	2.000
Alcoholism	0.022	4.559	2.236
Relaxin signaling pathway	0.023	6.438	2.000
VEGF signaling pathway	0.030	10.803	1.732
Mineral absorption	0.033	10.280	−0.577
Long-term depression	0.034	10.117	1.732
Long-term potentiation	0.041	9.105	1.732
Fc epsilon RI signaling pathway	0.042	8.977	1.732
Pancreatic cancer	0.049	8.278	1.732
Tight junction	0.050	4.721	2.000

**Table A4:** Gene Ontology (GO) terms of differential proteins between tongue-rolling and normal behavior cows.

GO Type	GO Terms	*p*-Value	Fold Enrichment
BP	Iron ion transport	<0.001	26.042
BP	Vesicle docking involved in exocytosis	0.001	53.455
BP	Vesicle-mediated transport	0.002	9.101
BP	Protein transport	0.002	6.490
BP	Antigen processing and presentation	0.002	42.319
BP	Protein stabilization	0.005	11.574
BP	Intracellular protein transport	0.005	7.024
BP	Endocytosis	0.006	10.498
BP	Antibacterial humoral response	0.007	24.182
BP	Neuron apoptotic process	0.008	21.609
BP	Negative regulation of cysteine-type endopeptidase activity	0.009	225.699
BP	Ras protein signal transduction	0.013	16.927
BP	Plasma membrane to endosome transport	0.014	135.419
BP	Response to dehydroepiandrosterone	0.014	135.419
BP	Response to 11-deoxycorticosterone	0.014	135.419
BP	Positive regulation of vasculogenesis	0.017	112.849
BP	Cell junction assembly	0.017	112.849
BP	Amyloid fibril formation	0.023	84.637
BP	Golgi organization	0.025	12.091
BP	Signal transduction	0.026	3.533
BP	Lipid transport	0.034	10.156
BP	Establishment of epithelial cell apical/basal polarity	0.034	56.425
BP	Nitric oxide biosynthetic process	0.034	56.425
BP	Regulation of endocytosis	0.043	45.140
BP	Response to progesterone	0.043	45.140
BP	Protein localization to plasma membrane	0.045	8.756
BP	Response to estradiol	0.046	42.319
CC	Golgi apparatus	<0.001	7.389
CC	Apical plasma membrane	<0.001	10.514
CC	Lipid droplet	<0.001	24.460
CC	Extracellular exosome	<0.001	19.353
CC	Autophagosome membrane	<0.001	34.364
CC	Early endosome	<0.00	10.462
CC	Endocytic vesicle	<0.001	29.977
CC	Phagocytic vesicle membrane	0.001	23.880
CC	GO:0055037~recycling endosome	0.002	15.831
CC	Plasma membrane	0.003	1.661
CC	Endoplasmic reticulum membrane	0.004	3.980
CC	Brush border membrane	0.004	32.021
CC	Golgi membrane	0.005	5.378
CC	Phagocytic vesicle	0.006	25.773
CC	Endomembrane system	0.006	10.436
CC	Cytoplasmic side of plasma membrane	0.014	16.511
CC	Dendritic spine	0.021	13.209
CC	Golgi lumen	0.022	88.058
CC	Lysosome	0.023	6.523
CC	Extracellular space	0.024	2.337
CC	Specific granule	0.025	78.274
CC	Late endosome	0.033	10.462
CC	Membrane raft	0.040	9.351
CC	Midbody	0.043	9.032
CC	Late endosome membrane	0.043	9.032
CC	Cytoplasm	0.043	1.535
CC	Phagophore assembly site membrane	0.049	39.137
MF	GDP binding	<0.001	68.597
MF	GTPase activity	<0.001	15.830
MF	GTP binding	<0.001	12.279
MF	G protein activity	<0.001	75.028
MF	Protein-containing complex binding	<0.001	15.733
MF	Oxidoreductase activity	0.002	9.475
MF	FAD binding	0.006	24.499
MF	Pyrimidine- and adenosine-specific: sodium symporter activity	0.011	171.492
MF	Protein-folding chaperone binding	0.015	15.590
MF	Uridine transmembrane transporter activity	0.017	114.328
MF	GTP-dependent protein binding	0.020	97.995
MF	Myosin V binding	0.034	57.164
MF	Protein homodimerization activity	0.040	3.802
MF	Myosin binding	0.048	40.351
